# Artificial Intelligence in Ocular Surface Oncology: A Narrative Review of Multimodal Imaging, Smartphone-Based Screening, and Clinical Decision Support

**DOI:** 10.3390/jcm15207785

**Published:** 2026-10-09

**Authors:** Francesco Cappellani, Marco Zeppieri, Antonio Longo, Cosimo Mazzotta, Irene Gattazzo, Fabiana D’Esposito, Caterina Gagliano, Matteo Capobianco

**Affiliations:** 1 Department of Medicine and Surgery, University of Enna “Kore”, Piazza dell’Università, 94100 Enna, Italy; francesco.cappellani@unikore.it (F.C.);; 2 Mediterranean Foundation “G.B. Morgagni”, 95125 Catania, Italy; 3 Department of Ophthalmology, University Hospital of Udine, 33100 Udine, Italy; 4 Department of Medicine, Surgery and Health Sciences, University of Trieste, 34129 Trieste, Italy; 5 Department of Ophthalmology, University of Catania, 95123 Catania, Italy; 6 Department of Ophthalmology, Ospedale Sant’Antonio, Azienda Ospedaliera, 35127 Padova, Italy; 7 Imperial College Ophthalmic Research Group (ICORG) Unit, Imperial College, 153-173 Marylebone Rd., London NW1 5QH, UK; 8 Eye Clinic, Catania University, Policlinico G. Rodolico, 95121 Catania, Italy

**Keywords:** artificial intelligence, machine learning, deep learning, ocular surface squamous neoplasia, conjunctival melanoma, conjunctival lymphoma, ocular surface tumors, anterior segment imaging

## Abstract

Ocular surface malignancies include ocular surface squamous neoplasia (OSSN), con-junctival melanoma, and conjunctival lymphoma. These rare disorders may threaten vi-sion and, in some cases, life. Early identification may be difficult because malignant le-sions can resemble benign ocular surface conditions. Artificial intelligence (AI), including machine learning and deep learning, has emerged as a potential aid to detection, classi-fication, and referral. This structured narrative review, organized with reference to SANRA principles, synthesizes PubMed/MEDLINE literature on AI applications in ocular surface oncology available through 29 August 2026. We examine slit-lamp photography, anterior segment optical coherence tomography, in vivo confocal microscopy, ultrasound biomicroscopy, and smartphone imaging. Deep-learning models have shown promising performance in selected binary OSSN tasks, whereas multiclass studies report lower sen-sitivity for rare malignancies such as conjunctival melanoma and lymphoma. Smartphone-based screening has also been evaluated in a prospective referral pathway, although the reference standard and verification process limit interpretation of popula-tion-level sensitivity. Dataset imbalance, image-acquisition variability, limited external and prospective validation, explainability, and workflow integration remain barriers to implementation. Future work should prioritize multicenter datasets, standardized acquisition, calibrated thresholds, and prospective human–AI evaluations. Current evidence supports further evaluation of AI as an adjunct for detection and referral, not as a re-placement for specialist assessment or histopathology.

## 1. Introduction

Artificial intelligence (AI) is progressively reshaping medical imaging and clinical decision support by enabling automated extraction, classification, and integration of complex diagnostic information [1]. Ophthalmology has emerged as one of the most active medical fields for AI development because of its imaging-intensive workflows, standardized image acquisition, and increasing availability of large digital datasets [2,3]. Deep learning (DL), in particular, can identify image patterns without requiring predefined handcrafted features and has achieved substantial diagnostic performance across several medical specialties [3,4]. In ophthalmology, the potential impact of AI extends beyond diagnostic classification to screening, referral prioritization, workflow optimization, teleophthalmology, and improved access to specialist care [5].

Despite rapid progress in retinal imaging and other high-volume ophthalmic applications, AI development in ocular oncology remains comparatively immature [6,7]. Recent reviews have identified a growing number of AI studies across retinoblastoma, choroidal tumors, ocular surface lesions, and other ophthalmic neoplasms, but the majority remain retrospective or developmental rather than prospectively deployed systems [6,7]. This translational gap is particularly relevant to ocular surface oncology, where malignant and potentially malignant lesions are uncommon, phenotypically heterogeneous, and frequently resemble benign conditions.

Ocular surface tumors encompass epithelial, melanocytic, lymphoproliferative, and other neoplastic processes involving the conjunctiva and cornea [8]. Clinical diagnosis traditionally relies on slit-lamp biomicroscopy supplemented by ancillary imaging and, when indicated, histopathological evaluation [9,10]. Ocular surface squamous neoplasia (OSSN) represents a spectrum ranging from intraepithelial dysplasia to invasive squamous cell carcinoma and constitutes the most common epithelial malignancy of the ocular surface [11,12]. Its biological heterogeneity is increasingly recognized at the genetic and epigenetic levels, although molecular characterization has not yet translated into routine AI-based precision oncology applications [13].

Conjunctival melanoma is considerably rarer but potentially lethal because of its capacity for local recurrence, regional lymphatic spread, and distant metastasis [14,15]. Early recognition and appropriate management are therefore essential, yet clinical differentiation from conjunctival nevus, melanosis, primary acquired melanosis/conjunctival melanocytic intraepithelial lesion, and other pigmented or amelanotic lesions may be challenging [14,15,16]. Conjunctival lymphoma represents another diagnostically important entity, most commonly arising from B-cell lineages and often presenting as a relatively nonspecific salmon-colored subepithelial lesion. For the purposes of this review, the term “ocular surface malignancies” is used operationally to encompass malignant and potentially malignant neoplastic disorders arising from or involving the conjunctival and corneal surface, with particular emphasis on OSSN, conjunctival melanoma, and conjunctival lymphoma. Diagnosis requires integration of ocular findings with histopathological, immunophenotypic, and systemic assessment [17,18].

These challenges have stimulated the development of non-invasive imaging techniques capable of providing structural information beyond conventional biomicroscopy [19]. High-resolution anterior segment optical coherence tomography (AS-OCT), in vivo confocal microscopy (IVCM), ultrasound biomicroscopy (UBM), and, more recently, optical coherence tomography angiography (OCTA) can characterize epithelial architecture, subepithelial morphology, cellular patterns, tumor depth, and vascular characteristics [20,21]. Contemporary AS-OCT studies have further expanded the recognizable imaging spectrum of OSSN, conjunctival melanoma, lymphoma, and their benign mimickers [10]. These modalities generate structured digital information that is particularly suitable for computational analysis.

AI applications specifically directed at ocular surface disease and ocular surface tumors have consequently expanded, and recent reviews have summarized developments in image classification and computer-assisted diagnosis [22,23]. However, the field is evolving rapidly, with several clinically relevant studies published in 2025 and 2026 that extend beyond conventional binary image classification [22,23]. These include multiclass slit-lamp models, self-supervised AS-OCT analysis, AI-assisted IVCM and UBM interpretation, low-shot learning for rare melanocytic tumors, and smartphone-enabled population screening. A clinically oriented appraisal is therefore needed to distinguish promising proof-of-concept results from technologies that are approaching meaningful clinical translation.

Accordingly, this narrative review critically examines the current role of AI in ocular surface oncology across the diagnostic pathway. Particular attention is given to the imaging substrate on which AI models are built; applications involving slit-lamp photography, AS-OCT, IVCM, and UBM; the specific limitations imposed by conjunctival melanoma and lymphoma as rare classes; and the emerging role of smartphone-based screening and clinical decision support. Finally, we discuss dataset quality, generalizability, explainability, prospective validation, and multimodal approaches that may determine whether AI can progress from isolated diagnostic models to clinically useful tools for early detection, triage, and specialist referral.

## 2. Materials and Methods

This article was designed as a structured narrative review. Its methodological organization was informed by the principles of the Scale for the Assessment of Narrative Review Articles (SANRA), including justification of the review topic, specification of its aims, description of the literature search, appropriate referencing, scientific reasoning, and presentation of relevant outcome data [24].

The PubMed/MEDLINE search covered publications available from database inception through 29 August 2026, with particular emphasis on studies published between 2019 and 2026; older publications were retained when they represented directly relevant foundational evidence. The final narrative synthesis included 83 publications. Because this study was designed as a structured narrative review rather than a systematic review, a PRISMA-style record of the number of citations retrieved and excluded at each screening stage was not prospectively maintained. Search concepts were combined around three principal domains: ocular surface oncology, artificial intelligence, and ophthalmic imaging. Search terms included combinations of “ocular surface tumor”, “ocular surface tumour”, “ocular surface neoplasia”, “ocular surface squamous neoplasia”, “OSSN”, “conjunctival melanoma”, “conjunctival lymphoma”, “conjunctival neoplasm”, “artificial intelligence”, “machine learning”, “deep learning”, “neural network”, “computer-aided diagnosis”, “slit-lamp”, “anterior segment optical coherence tomography”, “AS-OCT”, “high-resolution optical coherence tomography”, “in vivo confocal microscopy”, “IVCM”, “ultrasound biomicroscopy”, “UBM”, “optical coherence tomography angiography”, “OCTA”, “smartphone”, “telemedicine”, and “screening”.

Priority was given to peer-reviewed human studies directly evaluating AI-based diagnosis, classification, screening, or clinical decision support for ocular surface tumors. Recent systematic reviews, scoping reviews, and narrative reviews were used to establish the broader clinical and technological context. Studies describing the diagnostic characteristics of imaging modalities without an AI component were included when necessary to define the imaging substrate on which subsequent computational models were developed. Methodological publications addressing medical-AI reporting, validation, bias, multimodal learning, and clinical implementation were also considered.

Particular emphasis was placed on publications from 2019 to 2026. Older studies were retained when they represented foundational evidence concerning AS-OCT, IVCM, or other imaging characteristics of ocular surface tumors and remained directly relevant to the interpretation of contemporary AI models. Articles concerning exclusively posterior-segment, uveal, orbital, or eyelid tumors were not included in the disease-specific synthesis unless they contributed directly to a broader ocular oncology AI framework or to the interpretation of an integrated screening system.

For each relevant AI study, the review considered the target disease, imaging modality, dataset composition, reference standard, model architecture when reported, validation strategy, diagnostic performance, clinician comparison, external or prospective validation, and use of explainability methods. Particular attention was paid to rare-class representation, class imbalance, patient-level versus image-level validation, and evidence of real-world deployment. The literature search and study selection were independently conducted by two authors (F.C. and M.C.). Any disagreements regarding study eligibility or relevance were resolved through discussion and consensus. A formal domain-level risk-of-bias assessment was not prespecified because this work was designed as a structured narrative review and included heterogeneous study designs spanning image classification, diagnostic validation, screening, and clinical implementation. Nevertheless, to provide a more structured appraisal of the evidence, the principal AI studies were qualitatively evaluated for key risk-of-bias and applicability issues, informed by CLAIM and PROBAST+AI [25,26], with particular attention to dataset composition, reference standards, data partitioning, class imbalance, external validation, and clinical applicability.

Because the included studies were heterogeneous with respect to tumor type, image modality, sample composition, unit of analysis, reference standard, model architecture, and performance metrics, no quantitative meta-analysis was performed. Instead, findings were synthesized narratively with emphasis on clinical applicability and translational maturity. No new patient data were collected or analyzed for this review, and institutional ethical approval was therefore not required.

## 3. Current Evidence and Clinical Applications of AI in Ocular Surface Oncology

### 3.1. Clinical and Imaging Substrate for AI in Ocular Surface Oncology

The performance and clinical relevance of AI algorithms depend fundamentally on the quality and biological relevance of the images from which they learn [27]. In ocular surface oncology, conventional slit-lamp photography captures macroscopic features such as lesion pigmentation, vascularity, surface configuration, keratinization, feeder vessels, location, and extent, whereas cross-sectional imaging adds information that is not directly visible during clinical examination [10,28].

AS-OCT has become one of the most extensively characterized non-invasive modalities for ocular surface tumors [10]. Studies correlating AS-OCT with clinical and histopathological findings have demonstrated lesion-specific structural patterns across epithelial, melanocytic, and lymphoproliferative disorders [28,29]. Early ultra-high-resolution OCT studies established that imaging findings may closely correspond to histopathological architecture and can help refine the differential diagnosis of clinically ambiguous lesions [29,30]. In OSSN, the characteristic pattern includes a thickened, hyperreflective epithelium with an abrupt transition between normal and abnormal epithelium, whereas pterygium typically exhibits a relatively normal-thickness epithelium overlying hyperreflective subepithelial tissue [28,31]. HR-OCT can retain diagnostic utility even when OSSN coexists with scarring, limbal stem-cell deficiency, pterygium, or other ocular surface disorders that obscure the clinical appearance [32,33].

Nevertheless, AS-OCT is not equivalent to histopathology. In a prospective South African study including 182 conjunctival masses, OCT using an epithelial-thickness cutoff of 140 μm achieved a sensitivity of 87.2% and specificity of 75.6% for OSSN relative to histology, while image shadowing occurred in a substantial proportion of lesions [34]. Consequently, the concept of an OCT “optical biopsy” is clinically attractive but should be interpreted as an adjunctive rather than literal replacement for tissue diagnosis [29,35].

IVCM provides cellular-level information and has been investigated for both epithelial and pigmented lesions. In an early study of pigmented conjunctival tumors, confocal features showed encouraging discrimination of melanoma, although the cohort was small and the technology cannot substitute for histological characterization [36]. OCTA offers complementary vascular information. Studies of melanocytic conjunctival and iris lesions have demonstrated its technical feasibility, although vascular features alone were insufficient for reliable benign–malignant discrimination [37]. In OSSN, longitudinal OCTA imaging has demonstrated changes in vascular density and organization during topical therapy, suggesting a potential role for quantitative monitoring rather than established standalone diagnosis [21].

For lymphoproliferative disease, HR-OCT can demonstrate a relatively homogeneous hyporeflective subepithelial lesion with smooth borders and monomorphic dot-like infiltrates. However, overlap with benign reactive lymphoid hyperplasia may persist, and definitive classification continues to require tissue-based assessment [29,38]. Recent studies continue to characterize AS-OCT phenotypes across conjunctival tumors, but sample sizes remain modest [39]. These modality-specific phenotypes provide the biological and imaging foundation on which AI models can be trained; importantly, they also demonstrate why integration of multiple complementary modalities may ultimately be more useful than algorithms derived from a single image type. The principal imaging modalities considered in this review differ substantially in the type and resolution of information provided, equipment requirements, clinical availability, and scalability for AI applications; these characteristics are summarized in Table 1.

### 3.2. Slit-Lamp Photography and Deep-Learning Classification

Slit-lamp photography represents the most accessible source of digital data for AI because it reflects the examination already performed in routine ophthalmic practice and does not require additional specialized equipment [23,40]. Recent studies suggest that DL can extract diagnostically useful patterns from these photographs, although model performance varies considerably according to disease prevalence and class composition [40,41,42].

The most comprehensive recent multiclass study was reported by Sakata et al. in 2026 [40]. A YOLOv5-based model was developed using 1491 slit-lamp images spanning seven ocular surface conditions: nevus, limbal dermoid, mucosa-associated lymphoid tissue (MALT) lymphoma, OSSN, melanoma, pinguecula, and pterygium [40]. External validation involved 299 images and comparison with ophthalmologists of different experience levels. The overall model positive predictive value was 96.0%. However, aggregate performance concealed substantial differences among disease classes. Sensitivity was 0.991 for pterygium but only 0.681 for OSSN, 0.550 for MALT lymphoma, and 0.357 for melanoma. Similarly, the positive predictive value for melanoma was only 38.5%, despite an AUC of 0.966 [40].

This discrepancy is particularly important for interpreting AI studies in rare cancers. High overall accuracy or AUC may coexist with clinically inadequate sensitivity for the very malignancy the system is intended to identify. In the Sakata dataset, there were 1118 pterygium images but only 14 melanoma and 20 MALT lymphoma images, providing a clear example of the class-imbalance problem inherent to ocular surface oncology [40].

More focused binary models have reported higher results. Ramezani et al. evaluated 162 slit-lamp photographs, including 77 OSSN and 85 pterygium images, using automated segmentation and transfer learning. The model achieved an AUC of 0.98 and reported sensitivity, F1 score, and accuracy of approximately 94% using 10-fold cross-validation [41]. Rehman et al. subsequently evaluated 634 images comprising OSSN, other ocular surface lesions, and normal ocular surfaces. Among the architectures tested, MobileNetV2 achieved an average OSSN detection accuracy of 88.8%, sensitivity of 74%, and specificity of 96.25% [42].

Collectively, these studies demonstrate that slit-lamp-based AI can identify diagnostically relevant tumor morphology. However, binary OSSN-versus-pterygium discrimination represents a narrower task than real-world triage, where clinicians encounter a broad range of benign, premalignant, inflammatory, infectious, melanocytic, and lymphoproliferative lesions. The lower rare-class performance observed in multiclass settings therefore likely provides a more realistic representation of the challenges facing autonomous or semi-autonomous diagnostic systems [40,41,42].

### 3.3. AI Applied to AS-OCT, IVCM, and UBM

AI analysis of cross-sectional and cellular imaging offers the possibility of exploiting features that are less dependent on external appearance. The strongest evidence to date concerns OSSN [43,44,45].

Greenfield et al. developed an AS-OCT model using self-supervised pretraining with 105,859 unlabeled images from 5746 eyes, followed by supervised fine-tuning of a Vision Transformer classifier [43]. Evaluation was performed using 566 scans from 62 eyes in 48 patients with biopsy-confirmed diagnoses. At the scan level, the model achieved an accuracy of 90.3%, sensitivity of 86.4%, specificity of 93.2%, and AUC of 0.945 for differentiating OSSN from pterygium or pinguecula. Expert graders evaluating the OCT scans without accompanying clinical photographs or clinical data showed lower sensitivity and an AUC of 0.688 [43]. The comparison should therefore not be interpreted as demonstrating general superiority of AI over clinicians in routine practice, because the clinicians were deliberately deprived of information ordinarily incorporated into diagnosis.

AI has also been applied to IVCM. Kozma et al. analyzed 2774 IVCM images using ResNet50V2, YOLOv8x, and VGG19 architectures and incorporated data augmentation, few-shot approaches, and interpretability methods. Binary classification performance exceeded 97% accuracy across the reported models, whereas performance declined for more granular multiclass classification [44]. The study demonstrates the potential of cellular-resolution imaging for computational analysis but also reinforces the need for independent patient-level and multicenter validation before such performance estimates can be generalized.

UBM provides a different source of information, including lesion thickness, internal echogenicity, deep extension, and relationships with adjacent structures. In 2026, Ceylanoglu et al. evaluated a convolutional neural network using UBM images from 139 patients with biopsy-proven conjunctival lesions [45]. The model achieved a mean accuracy of 74.3%, sensitivity of 75.0%, specificity of 73.0%, and AUROC of 0.83. It significantly outperformed two ocular oncology fellows and showed borderline statistical differences compared with a senior ophthalmologist. Heatmaps suggested that lesion echogenicity contributed to model decisions [45].

These findings suggest that AS-OCT, IVCM, and UBM should not be regarded as interchangeable sources of AI data. AS-OCT primarily characterizes epithelial and subepithelial microarchitecture; IVCM captures cellular morphology; and UBM provides deeper structural information. Their complementary nature supports future multimodal rather than competitive use [43,44,45,46].

### 3.4. Conjunctival Melanoma, Lymphoma, and the Rare-Class Problem

Whereas multiple studies have addressed OSSN, the evidence supporting AI specifically for conjunctival melanoma and conjunctival lymphoma remains sparse [22,23,40,47]. This distinction is clinically important because it limits the extent to which results obtained in OSSN can be generalized to “ocular surface oncology” as a whole.

Yoo et al. specifically addressed the small-data problem in conjunctival melanoma using low-shot DL, although this study was published in 2021 [47]. Their dataset comprised 136 conjunctival melanoma images, 93 nevus or melanosis images, 75 pterygium images, and 94 normal conjunctival images. Generative adversarial networks and transfer learning were used to augment model development, with additional experimentation involving smartphone-acquired images of melanoma phantoms [47]. The study was important conceptually because it explicitly addressed scarcity of malignant examples rather than assuming that conventional large-dataset DL conditions were available.

The 2026 multiclass study by Sakata et al. provides more recent evidence, but simultaneously demonstrates the limitations of current datasets: only 14 melanoma and 20 MALT lymphoma cases were available, producing substantially lower disease-specific sensitivity and positive predictive values than for common benign classes [40]. No mature, dedicated AI system for conjunctival lymphoma with large external validation cohorts has yet emerged from the literature reviewed here. Because extranodal marginal zone lymphoma of mucosa-associated lymphoid tissue (MALT lymphoma) represents the predominant histologic subtype of conjunctival lymphoma, digital pathology may provide an additional future source of AI-derived information beyond ocular imaging alone. AI-based analysis of lymphoma histopathology has already been investigated for diagnostic classification and subtype recognition, including recent work specifically involving MALT lymphoma on hematoxylin and eosin-stained tissue. Integration of digital histopathology with slit-lamp, AS-OCT, or other ocular imaging could therefore represent a future multimodal approach linking non-invasive lesion characterization with tissue-level diagnosis. However, such integrated models have not yet been validated specifically for conjunctival lymphoma and should currently be considered a research direction rather than an established clinical application [48,49].

For these rare malignancies, synthetic augmentation, transfer learning, self-supervised learning, and foundation-model approaches may reduce the amount of labeled data required for model development [22,43,50,51]. However, they cannot fully substitute for diverse, correctly labeled examples of genuine disease. In particular, synthetic data cannot by itself establish clinical generalizability [50], and models intended for rare tumor detection must be evaluated at clinically realistic prevalence and against challenging benign mimickers.

### 3.5. Smartphone-Based Screening and Clinical Decision Support

The greatest potential public-health impact of ocular surface AI may arise not from replacing specialist diagnosis but from identifying patients who should reach specialist care. Smartphone photography is particularly attractive in this context because suspicious external ocular lesions can potentially be imaged without dedicated ophthalmic equipment [52,53].

A major translational step was reported by Wang et al. in *JAMA Ophthalmology* in 2026 [52]. The investigators developed and validated a DL system using multicenter slit-lamp images collected over 12 years, subsequently adapted it to smartphone-acquired images, and deployed the CaptureTumor platform in a nonrandomized multicenter clinical trial in China. The application incorporated AI-guided image acquisition, immediate malignancy risk stratification, and referral of high-risk participants. Media outreach reached 256,053 individuals, of whom 614 completed at-home self-screening. Twenty malignancies were histopathologically confirmed, including 19 newly diagnosed cases. In a separate prospective smartphone validation cohort, the model achieved an AUC of 0.905, whereas the subsequent population implementation analysis reported an AUC of 0.977, sensitivity of 89.3%, and specificity of 95.9% [52]. These estimates should be interpreted cautiously. Histopathological verification was primarily available for surgically managed participants, while the study used a hierarchical reference standard comprising histopathology, clinical diagnosis, or telemedicine assessment depending on the participant pathway. Consequently, the reported population-level sensitivity cannot be interpreted simply as the proportion of the 20 histopathologically confirmed malignancies detected, and the denominator underlying the reported 89.3% sensitivity is not clearly specified in the main report. This differential verification introduces potential verification bias, particularly because occult malignancies among nonreferred participants were not systematically excluded histopathologically. The higher population-level AUC (0.977) compared with the prospective smartphone validation AUC (0.905) also reflects different study cohorts and reference-standard procedures and should therefore not be interpreted as evidence that deployment itself improved intrinsic model discrimination.

This study differs fundamentally from retrospective image-classification experiments because the AI system was embedded into a complete pathway involving image acquisition, automated assessment, patient communication, and referral. It therefore offers a proof of concept for AI-supported case finding rather than merely algorithmic discrimination. However, its interpretation requires caution. The system covered several external ocular and periocular lesion categories rather than a narrowly defined cohort consisting only of conjunctival OSSN, melanoma, and lymphoma. Moreover, population participation, image acquisition quality, disease prevalence, demographic generalizability, and the ability to reproduce results outside the originating health system remain critical questions [52,53].

Accordingly, the most realistic near-term role for smartphone AI may be triage, not definitive diagnosis: identifying images that are suspicious, prompting timely ophthalmic assessment, and supporting referral pathways in areas where ocular oncology expertise is limited [52,53]. The principal studies directly evaluating AI-based classification, diagnosis, or screening of ocular surface tumors are summarized in Table 2.

### 3.6. From Diagnostic Classification to Clinical Decision Support

The clinical value of AI ultimately depends on whether diagnostic output changes patient management appropriately. This issue is particularly relevant in OSSN, where management has evolved from predominantly excisional strategies toward increased use of topical pharmacotherapy. A survey of corneal specialists documented a substantial rise in primary topical monotherapy between 2003 and 2022 [54]. Contemporary reviews describe interferon alfa-2b, 5-fluorouracil, and mitomycin C as established medical options, with treatment selection influenced by lesion characteristics, drug availability, toxicity, patient factors, and clinician preference [55]. A systematic review and meta-analysis found broadly comparable outcomes between topical pharmacotherapy and surgical excision within the available evidence, although the underlying studies were heterogeneous and limited [56]. Additional recent clinical series support 5-fluorouracil as a therapeutic option in selected OSSN patients [57].

For conjunctival melanoma, management is more complex and generally requires specialized ocular oncology assessment, histopathological characterization, local control strategies, and appropriate surveillance for regional or systemic disease [58,59]. AI classification cannot substitute for these processes. Similarly, suspected conjunctival lymphoma requires tissue diagnosis and systemic evaluation [17,18].

**Figure 1:**
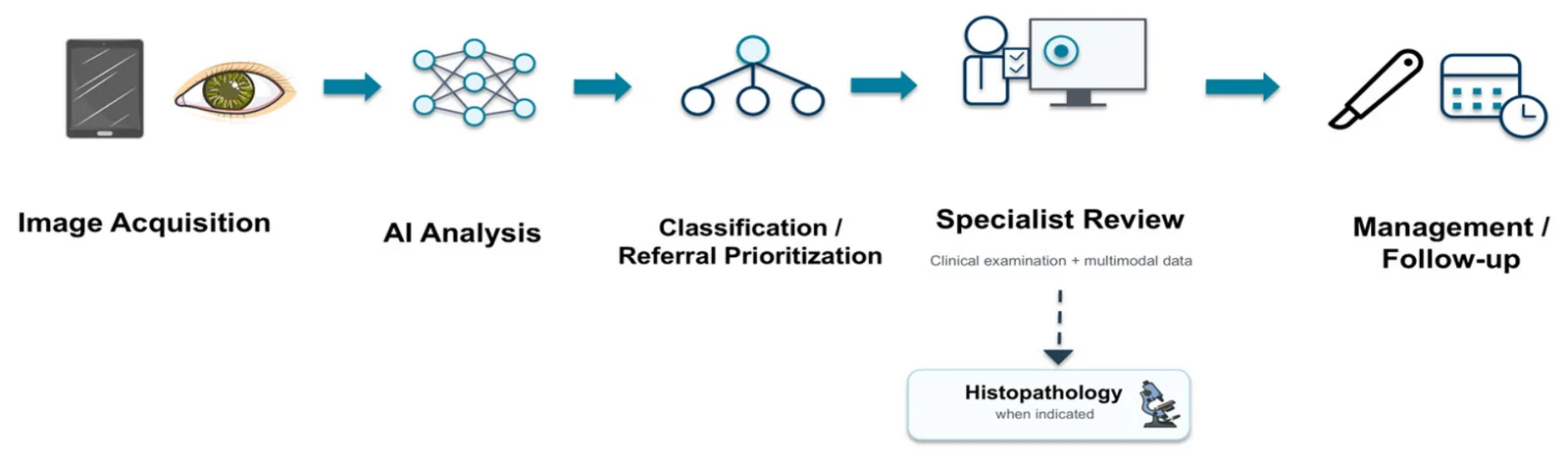
Conceptual framework for the integration of artificial intelligence across the ocular surface oncology pathway. Image(s) provided by Servier Medical Art (https://smart.servier.com), licensed under CC BY 4.0 (https://creativecommons.org/licenses/by/4.0/).

Consequently, current evidence supports AI primarily at the detection, differentiation, and referral stages of the ocular surface oncology pathway. Evidence that AI can independently select treatment, predict therapeutic response, or provide validated tumor-specific prognostication remains insufficient. Claims of “clinical decision support” should therefore presently refer to assistance with diagnostic categorization, prioritization, surveillance imaging, and referral rather than autonomous therapeutic recommendations. The current and potential integration of AI across the ocular surface oncology pathway is summarized in Figure 1.

## 4. Discussion

The available evidence indicates that AI has moved from theoretical applicability to demonstrable diagnostic performance in ocular surface oncology, particularly for OSSN. Recent studies show that DL can distinguish OSSN from common benign mimickers using slit-lamp photographs, recognize structural features on AS-OCT, classify IVCM images, and extract useful information from UBM. The smartphone-based clinical trial published in 2026 further demonstrates that AI can be incorporated into a real-world screening and referral pathway [40,41,42,43,44,45,46,47,52]. Nevertheless, the maturity of these applications differs substantially from the impression created by headline accuracy or AUC values.

A first major finding of this review is the marked disease imbalance of the evidence base. OSSN dominates current AI research, whereas conjunctival melanoma and particularly conjunctival lymphoma remain underrepresented. This imbalance is not incidental: it reflects the rarity of these diseases and therefore constitutes one of the fundamental methodological barriers to model development. The Sakata et al. study illustrates the problem particularly clearly. Although the multiclass model achieved high overall performance and high AUCs, sensitivity fell to 35.7% for melanoma and 55.0% for MALT lymphoma [40]. In a screening application, false-negative rates of this magnitude would be clinically consequential. Therefore, overall accuracy, weighted averages, or even high class-specific AUC values should never be interpreted in isolation. The Sakata study also highlights the importance of the operating threshold used to convert model probabilities into clinical classifications. A high AUC indicates good overall discrimination across possible thresholds but does not guarantee high sensitivity at the operating point selected for deployment. This distinction is particularly important for rare malignancies, in which the clinical cost of a false-negative result may justify selecting thresholds that favor sensitivity over specificity. Threshold selection should therefore be prespecified according to the intended clinical use, evaluated in representative validation cohorts, and accompanied by assessment of model calibration. In highly imbalanced datasets, calibration and prevalence-sensitive performance measures are particularly important because apparently strong discrimination may coexist with poor minority-class detection [60,61]. Cost-sensitive or class-specific thresholds may ultimately be more appropriate than a single uniform threshold when the consequences of missing melanoma or lymphoma differ substantially from those of generating additional benign referrals [60,61].

A second major finding is that the imaging modality determines the biological information available to an algorithm. Slit-lamp images provide clinically intuitive surface morphology and are scalable, inexpensive, and compatible with telemedicine. AS-OCT provides reproducible cross-sectional architecture and may better reveal epithelial transitions or subepithelial localization. IVCM offers near-cellular information but has a smaller field of view and greater acquisition dependence. UBM provides deeper structural information but lower image resolution than optical modalities [10,28,36,45]. The next logical developmental stage is therefore multimodal AI rather than increasingly complex algorithms applied to a single image source. Multimodal biomedical models can theoretically combine complementary imaging, clinical, demographic, histopathological, and molecular information, potentially improving robustness where any single modality is ambiguous [46]. Foundation-model approaches may further enable knowledge transfer across tasks and reduce dependence on large disease-specific labeled datasets [51].

However, multimodal models also introduce new technical and methodological challenges. Missing modalities, differences among imaging devices, acquisition protocols, variable image quality, and synchronization of patient-level information can all introduce bias. Moreover, a complex multimodal model may demonstrate superior discrimination while becoming more difficult to audit and reproduce. Future ocular surface AI research should therefore incorporate contemporary reporting frameworks. CLAIM provides specific recommendations for AI studies involving medical imaging [25], while TRIPOD+AI establishes updated reporting principles for prediction models irrespective of whether they use conventional regression or machine learning [62]. PROBAST+AI provides a complementary framework for assessing risk of bias and applicability [26].

A third issue concerns comparison between AI and clinicians. Several ocular surface studies have compared model performance with ophthalmologists and, in some settings, reported equal or superior algorithmic results. Such comparisons are informative but require careful methodological interpretation. Systematic evaluations across medical imaging have shown that many AI-versus-clinician studies have design or reporting limitations, and relatively few use robust external or prospective evaluation [63,64,65]. In the AS-OCT study by Greenfield et al., for example, clinicians interpreted isolated OCT scans without access to the slit-lamp photographs or clinical information that would normally inform their judgment [43]. The appropriate clinical question is therefore not simply whether an algorithm can “beat” an ophthalmologist on an artificial image-only task, but whether ophthalmologist plus AI improves diagnostic accuracy, efficiency, referral appropriateness, or patient outcomes compared with current practice.

Generalizability is another critical limitation. Variations in ethnicity, tumor prevalence, referral patterns, camera systems, OCT platforms, image compression, illumination, and clinical spectrum may cause dataset shift when models are transferred between institutions. Federated learning may offer a complementary strategy for improving multicenter model development and generalizability in this setting. By allowing participating institutions to collaboratively train models while retaining raw patient data locally, federated approaches can facilitate cross-institutional collaboration without requiring centralized data repositories. This may be particularly relevant for rare ocular surface malignancies, for which individual centers often have limited case numbers and data-sharing constraints can impede aggregation of sufficiently diverse datasets. Ophthalmic literature has identified federated learning as a potential approach for cross-institutional collaboration in rare diseases and for addressing privacy and regulatory barriers to data sharing. However, its utility in ocular surface oncology remains to be demonstrated in prospective multicenter studies [66,67]. Dataset shift is a recognized threat to medical AI performance [27,68,69], and a model that has undergone one external validation may still deteriorate as local populations, devices, or workflows change. Recurring local validation has therefore been proposed as a more appropriate paradigm than viewing external validation as a one-time event [69]. This concept is particularly relevant in ocular surface oncology because disease phenotype and prevalence may vary substantially across geographical regions and patient populations.

Explainability may facilitate clinical confidence but should also be interpreted carefully. Grad-CAM heatmaps and related visualization approaches used in recent ocular surface studies can demonstrate which image regions influence model output [42,44,52], while the IVCM study by Kozma et al. incorporated SHAP-based approaches [44]. Such visualizations are useful for identifying obvious shortcut learning—for example, attention directed to nonlesional image borders—but they do not prove that a model has learned a biologically causal feature. Explainability should therefore complement rather than replace rigorous validation.

Deployment also requires attention to the human factors surrounding AI. Successful clinical integration depends on workflow compatibility, liability, cybersecurity, privacy, interoperability, reimbursement, clinician trust, and patient acceptance [2,70,71]. Recent ophthalmic literature suggests generally favorable attitudes toward AI but also highlights concerns regarding privacy, trustworthiness, accountability, cost, and the need for appropriate education [71]. In oncology, these considerations are especially important because a false reassurance may delay diagnosis of a life-threatening disease, whereas excessive false-positive referral may overload specialist services and generate unnecessary biopsies and patient anxiety. These considerations also raise specific ethical issues in ocular surface oncology. Because the consequences of misclassification are asymmetric, false reassurance from an AI system may delay the diagnosis of a potentially life-threatening malignancy, whereas excessive false-positive classifications may expose patients to anxiety, unnecessary specialist referrals, and potentially avoidable diagnostic procedures. The marked underrepresentation of rare classes such as conjunctival melanoma and lymphoma also raises concerns regarding fairness, as apparently strong overall model performance may conceal substantially poorer performance for the patients with the least frequently represented diseases [40]. Transparent communication regarding when AI is being used, its intended role, and its limitations is therefore important, particularly when systems are deployed for screening or triage. Protection of ocular images and associated clinical information, appropriate governance of data used for model development, and equitable access to AI-enabled screening should also be considered [2,70,71]. Finally, clinical responsibility should remain clearly defined, with meaningful human oversight and the possibility for clinicians to override AI recommendations when they conflict with clinical findings. Regulatory requirements represent an additional barrier to clinical translation. AI systems intended to support diagnosis or referral may qualify as medical device software and must therefore satisfy jurisdiction-specific requirements for safety, clinical performance, risk management, and post-market surveillance. In the European Union, medical AI may fall within the Medical Device Regulation and, depending on its intended use and risk classification, may also be subject to the high-risk requirements of the EU Artificial Intelligence Act, creating overlapping obligations related to data governance, technical documentation, human oversight, accuracy, robustness, cybersecurity, and lifecycle monitoring. In the United States, AI-enabled medical devices are evaluated through established FDA medical-device pathways, while evolving guidance increasingly addresses lifecycle management and the control of planned algorithm modifications. These requirements are particularly relevant to ocular surface oncology because small rare-disease datasets, heterogeneous imaging devices, and limited prospective external validation may complicate demonstration of sufficiently robust clinical performance. Additional challenges include protection and lawful use of sensitive health data, accountability for erroneous AI-assisted decisions, surveillance for performance drift after deployment, and differences between regulatory frameworks across jurisdictions. Consequently, regulatory readiness requires not only high diagnostic accuracy but also transparent documentation, representative clinical validation, controlled model updates, cybersecurity safeguards, and continued post-market performance monitoring [72,73].

The Wang et al. trial provides an important model for the type of evidence now required [52]. Its principal contribution is arguably not simply the reported AUC, but the prospective integration of AI-assisted image capture, classification, and referral into a patient-facing pathway. Future studies should extend this approach using multicenter and multinational populations and report outcomes that are clinically meaningful: proportion of malignancies detected, false-negative referrals, time to specialist assessment, stage or lesion extent at diagnosis, unnecessary referral rates, biopsy yield, patient adherence, cost-effectiveness, and potential disparities in access. Cost-effectiveness is particularly important when considering AI-based screening for rare diseases. Because disease prevalence is low, even a highly discriminative model may generate substantially more false-positive referrals than true-positive detections, making economic value dependent not only on diagnostic accuracy but also on specificity, operating thresholds, the population screened, and the downstream cost of specialist assessment and confirmatory testing. Conversely, smartphone-based AI may reduce costs by providing scalable, decentralized triage and concentrating specialist resources on individuals at higher risk. These potential savings must be balanced against software and infrastructure costs, system integration, maintenance and updating, personnel training, confirmatory examinations or biopsies, and the consequences of false-negative results. Economic evaluations of AI-based ophthalmic screening have shown that cost-effectiveness is strongly context dependent and should incorporate implementation costs, patient adherence, referral pathways, and local healthcare-resource use [74]. Although Wang et al. demonstrated the feasibility of large-scale smartphone screening with relatively limited resource requirements, their study did not provide a formal long-term health-economic evaluation, and cost-effectiveness therefore remains an important endpoint for future prospective studies in ocular surface oncology [52].

Early-stage clinical AI evaluation can be structured according to DECIDE-AI [75], whereas interventional trial protocols and reports should follow SPIRIT-AI and CONSORT-AI recommendations [76,77,78]. For ocular surface tumors specifically, future validation datasets should be partitioned at the patient rather than image level; include clinically difficult mimickers rather than artificially clean controls; document the histopathological reference standard whenever available; report class-specific sensitivity, specificity, positive and negative predictive values, calibration, and confidence intervals; and include truly external testing on images acquired with different devices and by different operators. Several complementary study designs will therefore be required to move the field beyond proof-of-concept performance. First, prospective multicenter diagnostic-accuracy studies should enroll consecutive or clinically representative patients, use prespecified operating thresholds, maintain patient-level separation, and compare model predictions with an appropriate reference standard, including histopathology when clinically indicated. Second, external validation should deliberately span different institutions, geographical populations, imaging devices, and acquisition protocols to assess transportability and dataset shift. Third, prospective paired-reader or randomized human–AI studies should compare ophthalmologists working with and without AI assistance, rather than comparing algorithms with clinicians on isolated images, and should evaluate whether AI improves diagnostic accuracy, referral appropriateness, efficiency, or patient outcomes. Finally, pragmatic implementation studies are needed for smartphone and community-based screening pathways, with outcomes including false-negative malignancies, referral burden, time to specialist assessment, biopsy yield, adherence, cost-effectiveness, and disparities in access. In rare malignancies, enriched retrospective datasets may remain useful for early model development, but clinically meaningful validation should ultimately be performed under prevalence and workflow conditions that approximate real-world practice [75,76,77]. Several reviews published since 2022 have examined AI in ocular oncology from complementary perspectives. Koseoglu et al. reviewed applications across intraocular and ocular surface malignancies, with much of the available evidence centered on uveal melanoma [79], while Qurban and Cassidy focused more specifically on ocular adnexal tumors [80]. More recent reviews have addressed the field at different levels of breadth: Vempuluru and Kaliki surveyed AI across the full spectrum of ocular oncology and assessed the translational maturity of published models [6], whereas Sachdeva et al. systematically evaluated diagnostic performance across external, anterior-segment, and posterior-segment ocular tumors [7]. Almobayed et al. considered AI-based diagnostic guidance across both neoplastic and non-neoplastic ocular surface disorders [23]. The review by Ghanbari et al. is closest to the present work in disease scope, providing a structured overview of AI applications in ocular surface tumors based on literature searched through May 2025 [22]. The present review places greater emphasis on the clinical translation of AI specifically within ocular surface oncology, including the relationship between imaging modality and model performance, rare-class representation in conjunctival melanoma and lymphoma, external and patient-level validation, smartphone-based screening and referral, and the distinction between technical performance and clinically meaningful deployment. The later search window also incorporates studies published in 2026 that were unavailable to reviews with earlier search cutoffs, allowing these developments to be considered within the broader question of how AI may be integrated into real-world ocular surface oncology pathways.

Several limitations of the present review should also be considered when interpreting this synthesis. The literature search was restricted to PubMed/MEDLINE and did not systematically include engineering databases such as IEEE Xplore or Scopus, or preprint servers; consequently, relevant technical or recently disseminated AI studies may have been missed. The search included publications available through 29 August 2026, which defines the temporal boundary of the evidence considered. Direct comparison across studies is also constrained by substantial heterogeneity in tumor type, imaging modality, reference standard, dataset composition, and validation strategy, which may contribute to spectrum and verification bias. These sources of bias are well established in diagnostic-accuracy research [81] and remain relevant in contemporary ophthalmic AI studies [27]. Second, the rarity of several ocular surface malignancies results in small and markedly imbalanced datasets, creating potential representation bias and reduced performance for minority classes. Class imbalance is a recognized methodological concern in medical AI [82], and this remains evident in recent ocular surface oncology studies, including the markedly lower sensitivity for melanoma and MALT lymphoma reported by Sakata et al. [40]. From a clinical perspective, these findings argue against interpreting current AI outputs for rare ocular surface malignancies as definitive diagnostic results. In particular, a negative or low-risk model output should not be considered sufficient to exclude melanoma or lymphoma when clinical suspicion remains. Class-specific sensitivity, false-negative rates, calibration, and the operating threshold used for classification should be considered alongside global measures such as accuracy or AUC. At the present stage, AI outputs for rare malignancies are therefore best interpreted as adjunctive information that may support specialist review, rather than as a replacement for clinical assessment. Finally, the limited availability of external and prospective validation may result in optimistic performance estimates and limited transportability across institutions, devices, and patient populations. The importance of independent validation has long been recognized [83], while recent clinical-AI literature continues to emphasize the need for validation across local populations and changing clinical environments [69]. The future role of AI in ocular surface oncology is therefore likely to be integrated into existing clinical pathways as an augmented rather than autonomous system. At the community or primary-care level, smartphone-based platforms could support standardized image acquisition, automated image-quality assessment, and preliminary risk stratification, identifying patients who require ophthalmic evaluation rather than attempting to establish a definitive diagnosis. AI-generated risk estimates could then be incorporated into referral pathways, with suspicious or uncertain cases prioritized for specialist assessment. Within ophthalmology clinics, AI could function as a second-reader or decision-support tool applied to slit-lamp photography and complementary imaging, highlighting suspicious lesions, quantifying lesion boundaries or epithelial abnormalities, and facilitating comparison with previous examinations. At tertiary referral centers, multimodal systems could integrate clinical photographs, AS-OCT and other imaging modalities with demographic and longitudinal clinical information to support specialist assessment and surveillance. Importantly, clinically consequential decisions should remain subject to ophthalmologist review, with histopathological confirmation performed whenever required by current diagnostic practice. Integration into electronic health records, clearly defined referral thresholds, interoperability with existing imaging systems, and mechanisms for clinician override and performance monitoring will also be necessary for successful implementation.

## 5. Conclusions

This review aimed to critically examine the current applications, methodological limitations, and translational potential of artificial intelligence in ocular surface oncology, with particular attention to multimodal imaging, smartphone-based screening, and clinical decision support. Artificial intelligence is emerging as a potentially valuable adjunct in ocular surface oncology, but the strength of evidence differs markedly among tumor types and imaging modalities. The most developed applications concern OSSN, for which DL models using slit-lamp photography, AS-OCT, IVCM, and UBM have demonstrated encouraging diagnostic performance. By contrast, evidence for conjunctival melanoma is substantially more limited, and dedicated AI evidence for conjunctival lymphoma remains particularly sparse. Recent work has also shifted the field beyond retrospective image classification. Smartphone-enabled AI combined with automated image acquisition and referral pathways demonstrates the feasibility of extending tumor detection outside specialist ophthalmic settings. Nevertheless, high algorithmic accuracy should not be equated with clinical readiness. Rare-class underrepresentation, dataset shift, heterogeneous acquisition protocols, insufficient external and prospective validation, and uncertainty regarding integration into real-world workflows remain major barriers. Future research should prioritize multicenter and geographically diverse datasets, patient-level external validation, clinically realistic differential diagnoses, multimodal integration, transparent reporting, and prospective evaluation of human–AI workflows. Clinically meaningful endpoints should extend beyond AUC and accuracy to include false-negative malignancies, referral appropriateness, time to diagnosis, patient outcomes, accessibility, and cost-effectiveness. At present, AI should therefore be considered a tool to augment ophthalmic assessment, screening, and referral rather than replace ocular oncologists or histopathological diagnosis. Its most plausible future value lies in connecting accessible image acquisition with multimodal specialist assessment, thereby improving early recognition and routing of patients with potentially vision- or life-threatening ocular surface malignancies.

## Figures and Tables

**Table 1:** Comparison of imaging modalities relevant to artificial intelligence in ocular surface oncology.

Imaging Modality	Principal Information	Relative Spatial Resolution	Relative Cost/Equipment Burden	Clinical Availability	Main Advantages for AI	Principal Limitations
Slit-lamp photography	Surface morphology, pigmentation, vascularity, keratinization, feeder vessels, lesion location and extent	Moderate; macroscopic lesion-level imaging	Low–moderate	High in ophthalmic practice	Routinely acquired, scalable, relatively standardized, and suitable for teleophthalmology and image classification	Limited depth information; image quality and appearance depend on illumination, magnification, camera system, and operator
AS-OCT/HR-OCT	Cross-sectional epithelial and subepithelial architecture, lesion thickness and transition zones	High; micrometer-scale structural imaging	Moderate–high	Moderate–high in ophthalmic centers	Objective cross-sectional information; reproducible structural features; useful for differentiating epithelial from subepithelial lesions	Shadowing in thick or highly reflective lesions; limited cellular characterization; device-dependent acquisition
IVCM	Near-cellular morphology and microstructural features	Very high; near-cellular resolution	High	Limited; mainly specialized centers	Provides detailed cellular-level information that is not available with conventional photography or OCT	Small field of view, operator dependence, contact acquisition, and limited availability
UBM	Deep lesion structure, thickness, posterior extent, and relationship with deeper tissues	Lower than optical modalities	Moderate–high	Moderate; generally specialist-based	Greater tissue penetration and visualization of deeper lesion components	Lower spatial resolution than optical imaging; contact examination and operator dependence
OCTA	Lesion-associated vascular density, morphology, and organization	High for vascular structural information	High	Moderate; dependent on OCTA-capable systems	Provides quantitative, non-invasive vascular information and may support longitudinal monitoring	Vascular features alone may not reliably discriminate benign from malignant lesions; artifacts and acquisition variability
Smartphone photography	External lesion appearance and surface morphology	Variable; dependent on camera, optics, illumination, and acquisition technique	Low	Very high	Portability, scalability, remote acquisition, and potential for community or patient-led screening	Variable image quality, limited acquisition standardization, absence of cross-sectional information, and dependence on camera/device characteristics

Abbreviations: AI, artificial intelligence; AS-OCT, anterior segment optical coherence tomography; HR-OCT, high-resolution optical coherence tomography; IVCM, in vivo confocal microscopy; OCTA, optical coherence tomography angiography; UBM, ultrasound biomicroscopy.

**Table 2:** Principal studies evaluating artificial intelligence for ocular surface tumor detection, classification, and screening.

Study	Target and Imaging Modality	Dataset and Unit of Analysis	AI Approach and Data Partitioning	Reference Standard and External Validation	Main Reported Performance	Key Methodological and Applicability Considerations
Sakata et al., 2026 [40]	Multiclass ocular surface lesion classification; slit-lamp photography	1491 images/eyes across seven classes: nevus, limbal dermoid, MALT lymphoma, OSSN, melanoma, pinguecula, and pterygium. Rare classes included 14 melanoma and 20 MALT lymphoma cases.	YOLOv5; 5-fold cross-validation; separate external validation set of 299 images.	Diagnostic labels derived from the study registry; reference standard was not uniform across all lesion categories. External validation: yes.	Overall PPV 96.0%. AUC: OSSN 0.954 (95% CI, 0.933–0.975), MALT lymphoma 0.894 (0.794–0.993), melanoma 0.966 (0.919–1.000). Sensitivity: OSSN 68.1%, MALT lymphoma 55.0%, melanoma 35.7%.	External validation was performed, but marked class imbalance and very small melanoma and lymphoma samples limit applicability to rare malignancies.
Ramezani et al., 2025 [41]	OSSN vs. pterygium; slit-lamp photography	162 images from 162 patients: 77 OSSN and 85 pterygium. Unit: image/patient.	EfficientNet-B7-based segmentation followed by GoogLeNet classification; 10-fold cross-validation.	Clinical diagnosis; histopathologic confirmation was not reported. External validation: no.	AUC 0.98; sensitivity 94%; F1 score 94%; accuracy 94%. 95%	Relatively small dataset and internal cross-validation only. One image was reported per patient, reducing concern regarding within-patient image overlap.
Rehman et al., 2025 [42]	OSSN vs. other ocular surface disease vs. normal surface; slit-lamp photography	634 original images: 163 OSSN, 202 other ocular surface disease, and 269 normal images. Unit: image.	MobileNetV2, Xception, and DenseNet121; 85:15 train/test split and 5-fold cross-validation; data augmentation. Patient-level separation was not reported.	Diagnostic labels from the clinical dataset; histopathologic confirmation was not uniform. External validation: no.	MobileNetV2: mean accuracy 88.8%, sensitivity 74%, specificity 96.25%. 95%	Image-level partitioning and lack of reported patient-level separation introduce potential concern for data leakage and limit assessment of generalizability.
Greenfield et al., 2025 [43]	OSSN vs. pterygium/pinguecula; high-resolution AS-OCT	Pretraining: 105,859 unlabeled scans from 5746 eyes. Supervised dataset: 2022 scans from 523 eyes. Test set: 566 scans from 62 eyes/48 patients. Unit: AS-OCT scan.	Masked-autoencoder pretraining followed by supervised Vision Transformer; supervised data were separated at the patient level. Eyes used for self-supervised pretraining were excluded from the supervised dataset.	Biopsy-confirmed diagnoses in the test cohort. Independent external-center validation: no.	Accuracy 90.3% (95% CI, 87.5–92.6%); sensitivity 86.4% (81.4–90.4%); specificity 93.2% (89.9–95.7%); AUC 0.945 (0.918–0.972).	Patient-level partitioning and exclusion of pretraining eyes from the supervised dataset reduce leakage risk; single-center evaluation limits broader generalizability.
Kozma et al., 2025 [44]	OSSN-related classification; IVCM	2774 IVCM images from 97 patients, including OSSN, normal cornea, melanoma, pterygium, and keratitis. Unit: image, with additional cell- and patient-level analyses.	ResNet50V2, YOLOv8x, and VGG19; patient-specific allocation prevented overlap between training and validation datasets; augmentation, few-shot learning, and interpretability analyses were also performed.	Histopathologic confirmation for OSSN; expert-validated image annotations for feature-level analyses. External IVCM datasets were used for the symptom-level classifier, but not as independent clinical validation of the primary OSSN diagnostic task.	In the initial binary healthy versus OSSN-diagnosed IVCM image task, accuracy was 98% for ResNet50V2, 99% for YOLOv8x, and 97% for VGG19. 95%	Multiple classification tasks and levels of analysis were investigated; therefore, headline performance values are not interchangeable. Patient-specific partitioning reduced leakage risk.
Serbest Ceylanoglu et al., 2026 [45]	OSSN vs. benign conjunctival epithelial lesions; UBM	139 patients with biopsy-proven lesions; 582 UBM images, with approximately 3–8 images per eye. Unit: image.	Xception-based CNN; 5-fold cross-validation; data augmentation applied during model training. Patient-level separation was not reported.	Histopathology. External validation: no.	Accuracy 74.3 ± 3.9%; sensitivity 75.0 ± 8.6%; specificity 73.0 ± 11.5%; AUROC 0.83 ± 0.03. Values reported as mean ± SD; 95%	Histopathologic confirmation strengthens the reference standard; however, multiple images were obtained from individual eyes and patient-level partitioning was not reported.
Yoo et al., 2021 [47]	Conjunctival melanoma detection and four-class lesion classification; ocular surface photography	398 images: 136 melanoma, 93 nevus/melanosis, 75 pterygium, and 94 normal conjunctiva. Unit: image.	GAN-based augmentation and transfer learning; original images divided 70%/10%/20% into training/validation/test sets. GAN-generated images were used to augment the training dataset only.	Diagnostic labels from the source image dataset. External smartphone evaluation used 3D melanoma phantoms rather than patients.	MobileNetV2 with GAN augmentation: accuracy 87.5% for four-class classification and 97.2% for binary melanoma detection; 94.0% accuracy on smartphone-acquired phantom images. 95%	Small rare-disease dataset; smartphone external evaluation used phantoms rather than patients, limiting direct clinical applicability.
Wang et al., 2026 [52]	Malignant vs. benign external ocular lesions and multiclass screening; slit-lamp and smartphone photography	Multistage multicenter datasets. Prospective siteless screening: 614 participants; 535 participants/805 images included in the final image-analysis dataset after quality control. Unit: image for model evaluation and participant for the screening pathway.	CaptureTumor deep-learning system; retrospective multicenter external testing, prospective slit-lamp testing, smartphone adaptation, and prospective siteless clinical implementation.	Specialist clinical evaluation with histopathology for surgically excised lesions and confirmed malignancies. External validation: yes; multicenter and prospective.	Prospective smartphone AUC 0.905 (95% CI, 0.837–0.973). Population implementation: AUC 0.977 (0.964–0.990), sensitivity 89.3% (86.7–91.9%), specificity 95.9% (94.2–97.6%).	Strong real-world validation, although only 20 malignancies were histopathologically confirmed and the system addressed a broader external ocular/periocular lesion spectrum rather than ocular surface malignancies alone.

Abbreviations: AI, artificial intelligence; AS-OCT, anterior segment optical coherence tomography; AUC, area under the receiver operating characteristic curve; CI, confidence interval; IVCM, in vivo confocal microscopy; MALT, mucosa-associated lymphoid tissue; OSSN, ocular surface squamous neoplasia; PPV, positive predictive value; UBM, ultrasound biomicroscopy.

## Data Availability

No new data were created or analyzed in this study. Data sharing is not applicable to this article.
